# Reply to Rockey et al., “Genomics and Chlamydial Persistence *In Vivo*”

**DOI:** 10.1128/mBio.02957-19

**Published:** 2019-12-17

**Authors:** Deborah Dean, Noa Ziklo, Naraporn Somboonna, Jung Hyuk Suh, Thomas Ferrin

**Affiliations:** a Center for Immunobiology and Vaccine Development, University of California San Francisco Benioff Children’s Hospital Oakland Research Institute, Oakland, California, USA; b Department of Bioengineering, University of California Berkeley and University of California San Francisco Joint Graduate Group, Berkeley and San Francisco, California, USA; c Department of Medicine and Pediatrics, University of California, San Francisco, California, USA; d Department of Pharmaceutical Chemistry, University of California, San Francisco, California, USA

**Keywords:** *Chlamydia trachomatis*, indole, interferon gamma, sexually transmitted diseases, *trpA*, tryptophan synthase

## REPLY

In addressing the letter to the editor by Rockey et al. (1), it is important to correct the statement “…patients can be colonized by C. trachomatis.” Chlamydia trachomatis does not colonize humans but infects them.

The authors’ earlier paper (2) described five patients that had apparent reinfection and/or persistent infection over a few years despite antibiotic treatment. On the basis of whole-genome sequencing (WGS), they concluded “…pathogen mutational strategies are not important in persistence of this pathogen in patients” (2). Our conclusion (that the clinical F strains we isolated yearly for 4 years from a female patient were associated with persistent infection) was based on a number of analyses, not just WGS (3). We examined growth characteristics in tissue culture of the clinical F strains by transmission electron microscopy (TEM), noting a persistent phenotype with aberrant bodies similar to the TEM of cervical tissue from a patient in New Orleans infected with a clinical E strain (4). In comparison with urogenital reference F and ocular reference A strains, we also examined structural protein features of the tryptophan operon; utilization of indole for tryptophan synthesis after tryptophan starvation *in vitro*; *trpA* and *trpB* expression levels under various conditions; and intracellular tryptophan levels. In these respects, our research design and analytical approach were quite different from theirs. Further, our findings dispute their conclusion.

Suchland et al. (2) did not find mutations in the *trpA* gene that were similar to ours or mutations in the rest of the operon (that includes the *trpR* and *trpB* genes) or in any read sets for the five strains. This is not surprising since their sample size was small. Additionally, none of their apparent persistent infections were with strain F. It is possible that there are F strains in the Seattle area that persist in patients and have similar *trpA* mutations but have not yet been discovered. More importantly, the lack of *trpA* mutations simply points to the fact that there are other host/pathogen mechanisms that also participate in the persistence phenotype. These mechanisms include, but are not limited to, various interferon gamma levels, antimicrobial pressure, and lack of host nutrients that are required for growth (e.g., iron). Our findings do not preclude the existence of these or other mechanisms.

Finally, we are perplexed by the last paragraph in the letter. Our paper does not propose a causal effect for a single “*in vitro* property” that would explain all C. trachomatis persistent infections. Our conclusions were based on the collective findings of the longitudinal *in vivo* phenotype in the patient and the multiple characteristics of the C. trachomatis F strains that we outlined above and analyzed and discussed in our paper (3). At the end of the abstract, we state, “Our data indicate that emergent mutations in the tryptophan operon, which were previously thought to be restricted only to ocular *Ct* [C. trachomatis] strains, likely resulted in *in vivo* persistence in the described patient and represents a novel host-pathogen adaptive strategy for survival” (3). As more C. trachomatis genomes are sequenced prospectively from different patient populations, we have the opportunity to detect novel mutations and begin to characterize these strains and examine their biological relevance for disease pathogenesis, including persistence.
